# Parks, Recreation, and Public Health Collaborative

**DOI:** 10.4137/EHI.S1017

**Published:** 2008-12-03

**Authors:** Judy Kruger

**Affiliations:** Division of Nutrition, Physical Activity and Obesity, Centers for Disease Control and Prevention, Atlanta, Georgia, U.S.A

**Keywords:** physical activity, collaboration, surveillance

## Abstract

The primary goal of many park and recreation agencies is to provide resources and programs that improve quality of life for the community. Increasing physical activity is one aspect of this agenda. Promoting physical activity is a public health goal; however, increasing population-level physical activity will require access to places for physical activity (e.g. parks). Practitioners and policy makers need more information to document the roles that parks and recreation facilities play to promote physical activity and contribute to public health. A working group of approximately 20 professionals experienced in data collection came together to discuss the needs for better surveillance and measurement instruments in the fields of parks, recreation, and public health. The working group made two major recommendations: (1) the need for collaborative research and data sharing, and (2) the need for surveillance measures to demonstrate the amount of health-related physical activity acquired in the park setting.

## Introduction

Parks play an important role in disease prevention by promoting physical activity. Objective 22–2 of the U.S. Department of Health and Human Services (DHHS) *Healthy People 2010* (HP2010) national health objectives describes the need to increase the proportion of adults who engage in regular, moderate, or vigorous-intensity physical activity to 50% or more (USDHHS, 2008). HP2010 suggests that the presence of parks may help people reach this target (USDHHS, 2008). Parks are an important community resource that can help people improve their health (Bedimo-Rung et al. 2005) and provide a safe place where people can walk, socialize with friends, or engage in team sports. Although, only one-third of the population exercises in a park setting (Cordell et al. 1999), recent findings showed that the number of people participating in outdoor recreation grew 4% from 1999 to 2008 (Cordell et al. 2008). An increase in surveillance efforts is one recommended strategy to monitor the availability and use of the park setting, as well as the frequency of park-based physical activity. Better surveillance of physical activity in the park setting is necessary to determine trends which guide public health policy and initiatives, and has significant implications for both private and public providers of recreation opportunities. This paper highlights the importance of collaborative partnerships to advance the surveillance of physical activity in the park environment.

Although the field of recreation and leisure studies has a long history assessing the park environment, more information that documents the contribution of parks and recreation to public health outcomes is needed. Recreation activity rates may be underreported because of a reliance on measures that focus only on the frequency and type of leisure participation, instead of measures that capture the amount of physical activity needed to promote health benefits. Adding surveillance questions that measure the intensity level of physical activity may provide the evidence relating park-based activity and health outcomes. A study by Hoehner and colleagues which examined how park use was related to meeting the public health recommendations of moderate-intensity activity 5 times per week, ≥30 minutes per activity, or vigorous-intensity activity 3 times per week, ≥20 minutes per activity (Pate et al. 1995) found that people who used a park ≥10 days per month were 3.4 times more likely to meet public health recommendations compared to non-park users (Hoehner et al. 2005). Agencies that incorporate unified methods and questions with proven psychometric properties can help strengthen advocacy opportunities, resource allocation, and the documentation of health-enhancing physical activity in the park setting.

Collaborative research among the fields of parks, recreation, and public health has formally started through a Memorandum of Understanding. A collaboration between the National Recreation and Park Association (NRPA) and the U.S. Department of Health and Human Services (DHHS) began in 2002 and was renewed and expanded in 2008 as part of a strategic partnership. The purpose of this partnership was to improve the health of the population by encouraging physical activity, reducing the numbers of overweight and obese people, and improving the health of communities through programs, products, and services. By working together, these federal agency partners affirmed the need to create a strong base of evidence from which to advocate collectively for policy change. One of the programs supported by this collaboration was the 2006 Cooper Institute symposium titled, “Parks, Recreation, and Public Health: Collaborative Frameworks for Promoting Public Health,” which highlighted the need to advance cooperation between parks, recreation, and public health researchers and practitioners. The 2006 Cooper symposium resulted in a supplement in the *Journal of Physical Activity and Health* with articles by authors from multidisciplinary fields such as epidemiology, leisure studies, recreation, and urban planning (Ainsworth et al. 2007; Buchner and Gobster, 2007; Librett et al. 2007). During this symposium, researchers and practitioners came together to form a collaborative working group named, “Parks for Physical Activity Research Consortium” (P-PARC). P-PARC expands surveillance of physical activity participation in outdoor recreation environments and promotes active visits in the park setting.

Important issues and concerns raised at the Cooper symposium made it possible for parks, recreation, and public health professionals to recommend how to gather the necessary evidence that links park-based activity to health-related physical activity levels. The dearth of cross-field research and data sharing has created a need for a cost-effective and time-efficient method for the collection of baseline estimates. The use of existing physical activity questions, such as those found in the Behavioral Risk Factor Surveillance System (BRFSS) that ask about the frequency (i.e. days per week) and duration (i.e. minutes per day) of moderate and vigorous-intensity physical activity (CDC, 2007), could be added to existing park and recreation survey instruments to link physical activity levels necessary to achieve health outcomes. The validity and reliability of the BRFSS physical activity questions (Yore et al. 2007) suggest that these questions are suitable to classify adults by activity status. Since 1994, the National Survey on Recreation and the Environment (NSRE) collects data on the number of people who participate in outdoor recreation activities, but does not assesses leisure-time physical activity in terms of frequency and duration. Adding the BRFSS questions to the NSRE instrument would help to gauge park users overall activity level.

Public health surveillance efforts are tied to the measurable health impacts of more than 900 HP2010 objectives and sub-objectives (USDHHS, 2008), and the analytic framework developed for HP2010 allows comparable measurement of health among population groups, over time, and across indicators (Keppel et al. 2004). As previously mentioned, physical activity objective 22–2 of HP2010 emphasizes the importance of engaging in regular physical activity at a dose that is likely to be adopted and maintained throughout one’s lifetime to confer health benefits. This objective intends to quantify the activity reported, equivalent to brisk walking or higher, for comparison to meeting the objective (i.e. meets or does not meet the objective). Because the HP2010 objectives are national, achievement of objectives depends on the ability of agencies at all levels of government to collect data. Consistent questions and data collection methods are used to compare local and state health department data to national data. It is possible to observe trends in the rate of physical activity participation because public health agencies have collected data consistently over the years; this is a benefit of using the established objective 22–2. The HP2010 database (http://wonder.cdc.gov/data2010) also provides state-specific estimates of physical activity levels across the United States. States can compare their individual progress to that of other states, a benefit to evaluating this objective.

An alternative to adding BRFSS physical activity level questions to an existing park and recreation survey instrument, is to inquire about park-based physical activity with new questions. Developing new surveillance questions is a costly and long-term process requiring both qualitative (e.g. focus groups, cognitive testing) and quantitative (e.g. reliability and validity testing) assessments. However, the development of a surveillance measure specific to the park setting which demonstrates health-enhancing physical activity as an outcome would be useful to establish common evidence in the fields of parks, recreation, and public health. Developing questions that elicit accurate reporting of physical activity is complicated because respondents often have difficulty recalling the details of participation in the various types of activity, including the intensity, duration, and frequency of activity (Durante and Ainsworth, 1996). New questions that ask respondents how much of their park visit involved physical activity are needed in order to understand how much physical activity people engage in during their park visit.

## Conclusion

To further understand how much physical activity people are obtaining in a park for health benefits, surveillance of park-based physical activity is a recommended strategy. Collaborative research to develop surveillance measures and data sharing may help to show the potential impacts of the outdoor recreational environment on physical activity levels. Using consistent surveillance questions across all levels of government and a robust methodology may lead to higher reported rates of physical activity. However, parks, recreation, and public health professionals need to do more than show an increase in park visits over time—they need to prove that park visits have some measurable benefit to health or quality of life.
